# Chest radiography evaluation in patients admitted with confirmed COVID-19 infection, in a resource limited South African isolation hospital

**DOI:** 10.4102/sajr.v26i1.2262

**Published:** 2022-01-17

**Authors:** Sereesh Moodley, Tanusha Sewchuran

**Affiliations:** 1 Department of Radiology, School of Clinical Medicine, Grey’s Hospital, University of KwaZulu-Natal, Pietermaritzburg, South Africa

**Keywords:** chest radiography, chest X-ray, SARS-CoV-2, COVID-19, chest X-ray scoring system, *Brixia* scoring system, resource limited setting, South Africa

## Abstract

**Background:**

Severe acute respiratory syndrome coronavirus 2 (SARS-CoV-2) and the subsequent global outbreak (coronavirus disease 2019 [COVID-19]) was declared a public health emergency in January 2020. Recent radiologic literature regarding COVID-19 has primarily focused on Computed Tomography (CT) chest findings, with chest radiography lacking in comparison.

**Objectives:**

To describe the demographic profile of adult patients with COVID-19 pneumonia requiring hospital admission. To describe and quantify the imaging spectrum on chest radiography using a severity index, and to correlate the severity of disease with prognosis.

**Method:**

Retrospective review of chest radiographs and laboratory records in patients admitted to a South African tertiary hospital with confirmed COVID-19 infection. The chest X-rays were systematically reviewed for several radiographic features, which were then quantified using the *Brixia* scoring system, and correlated to the patient’s outcome.

**Results:**

A total of 175 patients (mean age: 53.34 years) admitted with COVID-19 were included. Ground glass opacification (98.9%), consolidation (86.3%), and pleural effusion (29.1%) was commonly found. Involvement of bilateral lung fields (96.6%) with no zonal predominance (61.7%), was most prevalent. Correlation between the *Brixia* score and outcome was found between severe disease and death (odds ratio [OR]: 12.86; 95% confidence interval [CI]: 1.58–104.61). Many patients had unknown TB (71.4%) and HIV (72.6%) statuses.

**Conclusion:**

In this study population, ground glass opacification, consolidation, and pleural effusions, with bilateral lung involvement and no zonal predominance were the most prevalent findings in proven COVID-19 infection. Quantification using the *Brixia* scoring system may assist with timeous assessment of disease severity in COVID-19 positive patients, as an overall predicator of clinical outcome.

## Introduction

Severe acute respiratory syndrome coronavirus 2 (SARS-CoV-2) emerged in Wuhan, Hubei province, China in December 2019.^1^ Coronaviruses can cause multi-system infections in various animals; and mainly affects the respiratory tract in humans.^1^ The resultant pneumonia resembles SARS and Middle East respiratory syndrome (MERS). The genome of SARS-CoV-2, however, differs from SARS and MERS, and therefore this virus may lead to unconventional clinical presentations, with differing imaging findings.^2^ The subsequent global outbreak (coronavirus disease 2019 [COVID-19]) was declared a public health emergency internationally by the World Health Organization (WHO) on 30 January 2020.^3^ As of 03 December 2021, South Africa’s total confirmed cases were 3 004 203 with 89 944 deaths, according to statistics from the National Institute for Communicable Diseases (NICD).

Whilst a portion of patients infected with COVID-19 may be largely asymptomatic, the predominant clinical manifestation of COVID-19 is a lower respiratory tract infection which is evident in 17% – 29% of infected patients. The mortality rate is estimated at approximately 2.3%.^4^ It is suggested that COVID-19 is more likely to infect older adult males, especially when chronic comorbidities and weakened immunity are present. Weakened immunity further predisposes affected patients to co-infections with bacteria and fungi.^1^

COVID-19 can be specifically detected in respiratory secretions or plasma samples by reverse transcription polymerase chain reaction (RT-PCR).^5^ The RT-PCR testing is, however, limited as a diagnostic tool; when the viral load is low then detection rates are also low, thus leading to false negative results.^6^ Many studies have proposed chest CT as a routine imaging tool for COVID-19 pneumonia diagnosis, as it is quick and relatively easy to perform.^7^ The imaging findings of pneumonia caused by COVID-19 may, however, present its own diagnostic dilemma because of a potential overlap with other viral pneumonias, especially Pneumocystis Jirovecii Pneumonia (PJP) in our immunosuppressed population. Despite this, COVID-19 CT chest imaging has been proven beneficial particularly in symptomatic patients with negative RT-PCR tests, in patients with low viral loads, as well as in patients in the early stages of infection.^2^ Furthermore, RT-PCR only permits the diagnosis of a positive result, and as such the severity of COVID-19 disease as well as disease progression cannot be ascertained. This is where CT imaging has been shown to be beneficial and particularly assistive with disease progression.^6^

In most RT-PCR positive presumed symptomatic patients, CT examinations depict a typical pattern with a sensitivity of 97.0%.^8^ Typical CT features include ground glass opacities which may be accompanied by peripheral consolidation noted in proximity to visceral pleural surfaces, including fissures and usually with a multifocal bilateral distribution. Subpleural sparing can be visualised.^9^ One study demonstrated 30.2% of patients with single lobe involvement, whilst 44.4% of patients had all lobes involved.^10^

Consolidation, air bronchograms, ‘white lung’ appearance, and pleural effusions were more frequently seen in patients with severe disease. In these patients, a ‘crazy-paving’ pattern, linear densities, bronchiectasis, nodules, and tree-in-bud opacities were also more frequent.^2^ Several standardised reporting lexicons have been developed in an attempt to efficiently organise and grade CT imaging findings. Examples of these grading systems are CO-RADS and COVID-RADS.^8,9^ High incidences of thrombotic events (especially pulmonary embolism) have been found in intensive care unit (ICU) patients, with the European Society of Cardiology (ESC) recommending that CT pulmonary angiography (CTPA) be performed to exclude the presence of a pulmonary embolus when unenhanced CT findings cannot explain the severity of respiratory failure.^11^

Recent radiologic literature regarding COVID-19 has primarily been focused on CT chest findings, with information pertaining to chest radiography lacking in comparison. Chest radiography, especially when performed on portable machines, is considered to be a viable alternative as they are thought to pose a lower risk of cross infection and can be performed at the bedside. Chest radiography is currently deemed to be a useful first line triage tool for COVID-19, as well as an adjuvant to CT chest imaging, with a 69% sensitivity rate for chest X-rays (CXRs) in COVID-19, as reported by Wong et al.^12^

The most common feature of COVID-19 pneumonia detected on chest radiography is consolidation, with ground glass opacities being a close second. Patterns of peripheral, lower zone and bilateral distributions were predominant. Pleural effusions were seldom seen. Proposed severity scores peaked at 10–12 days from the onset of symptoms.^12^ The grading of CXRs can be done using a radiographic severity index, the *Brixia* score, which is a semi-quantitative assessment of lung disease in COVID-19 infection that ranks pulmonary CXR findings using an 18-point severity score, according to the characteristics and extent of lung abnormalities.^13,14^ This has proven to be a beneficial prognostic indicator in some European countries.^14^ Studies focusing on chest radiography findings of COVID-19 pneumonia provided little information regarding the presence of underlying pulmonary sequelae of other common infectious diseases such as tuberculosis (TB) and HIV. Features of superimposed infection (generally pleural effusions and lymphadenopathy) have also been described.^4^

This study aims to retrospectively analyse and quantify chest radiograph findings of confirmed COVID-19 patients admitted to a South African isolation hospital, where concomitant pulmonary manifestations of infectious diseases such and HIV and TB may pose an additional diagnostic dilemma.

The objectives of the study were: to describe the demographic profile of COVID-19 patients requiring admission during the first and second waves of the pandemic, dated from March 2020 to December 2020; to describe and quantify the imaging spectrum seen on chest radiography in admitted COVID-19 patients with the aid of a data collection tool; to evaluate for the presence of other co-morbidities, specifically HIV and TB on chest radiography; and to correlate the severity of disease, as determined by the *Brixia* scoring system, to the patients’ prognosis.

## Method

### Study design and participants

This was a single-centre, retrospective study conducted at a tertiary level South African isolation hospital. A review of chest radiographs as well as patient records was performed on patients with confirmed COVID-19 infection, who required hospital admission at Grey’s Hospital, Pietermaritzburg between March 2020 and December 2020.

All adult inpatients (> 18 years) admitted at Grey’s Hospital, Pietermaritzburg with a positive RT-PCR COVID-19 nasopharyngeal swab who received CXRs were included in the study.

### Data collection

All COVID-19 positive patients admitted during the stipulated time, who underwent CXRs were recorded on a database using the picture archiving and communications system (PACS). Epidemiological data was collected from this database, which included age and sex. Confirmation of positive COVID-19 infection, as well as associated medical co-morbidities, such as the HIV status and TB infection, was performed using the National Health and Laboratory Services (NHLS) system. Outcomes data was collected from the COVID-19 mortality database and correlated to discharge data from the hospital isolation wards. All data was then recorded on a standardised data collection form created by the authors.

Digital chest radiographs were acquired by a portable X-ray unit (AXIM Roller 30 portable unit) in various isolation wards in the hospital. The CXRs were evaluated by a consultant radiologist with specific thoracic imaging sub-specialty training and 10 years of experience, and a radiology registrar with two years of experience, with the aid of a data collection sheet. The reporting radiologists were blinded to the clinical (HIV and TB statuses) and prognostic (de-isolated or demised) data of the study population. Where there was disagreement between the reporting radiologists, a mutual consensus was reached and this value was used for analysis.

**FIGURE 1:**
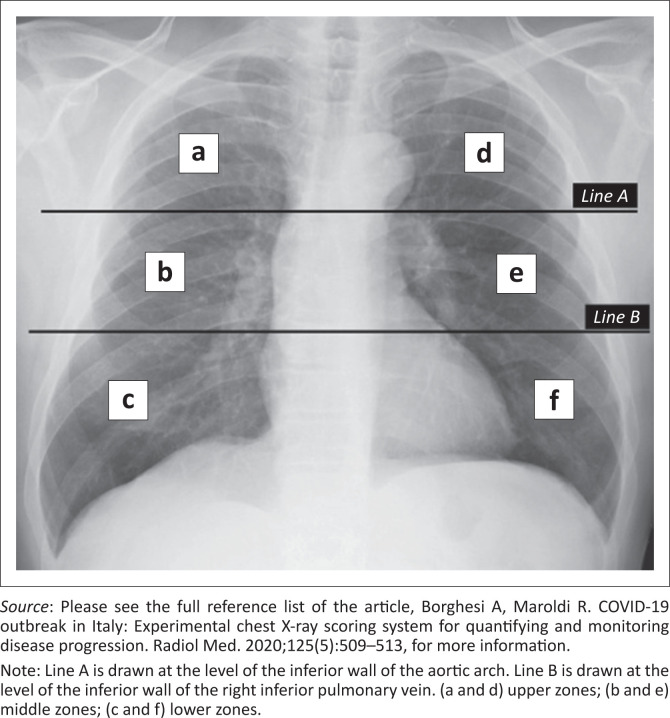
Division of lungs into six zones on frontal chest radiograph.

Chest X-rays were reviewed for several radiographic features, including ground-glass opacities, consolidation, bronchiectasis, cavitation, lymphadenopathy and masses/nodules as defined by the Fleischner Society glossary of terms.^15^ The distribution of lung changes was categorised into: upper zone, lower zone, or no zonal predominance; unilateral or bilateral lung involvement; as well as the number of pulmonary lobes involved. The presence of pleural effusions, volume loss, and probability of TB (e.g., cavities), was also documented. These findings were then quantified using the *Brixia* score.^13,14^ The *Brixia* scoring system involves two steps for image analysis. The first step involves dividing the lung on a frontal CXR into six zones namely upper zones (a and d), middle zones (b and e), and lower zones (c and f) (see Figure 1).^13^ The second step involves assigning a score (from 0 to 3) based on the lung abnormalities.^13^ A score of 0 indicates no lung abnormalities; a score of 1 indicates interstitial infiltrates; a score of 2 indicates interstitial and alveolar infiltrates with interstitial predominance; and a score of 3 indicates interstitial and alveolar infiltrates with alveolar predominance.^13^ The scores are then tallied and a final score (out of 18) is determined.^13^ Finally, a comparison with previous baseline CXRs as well as repeat CXRs, if available on the PACS, was also assessed. The patient’s CXR with the highest *Brixia* score was used for final analysis and the date of this X-ray was recorded for correlation with mortality data.

## Statistical analysis

Data were analysed using Stata version 15 statistical software. For continuous variables like age, the mean, median, and range were calculated. The results were recorded as frequencies and percentages for categorical variables. With regards to comparing the four categories of the *Brixia* score, the categories are on an ordinal scale and overall comparisons were performed using Kruskal-Wallis, followed by Dunn tests for pairwise comparisons. Age was categorised into three age groups and Chi- square tests were used to compare outcome. Sex comparisons were made using Chi-square tests. Statistical significance was set at *p* < 0.05. The odds ratio (OR) and 95% confidence interval (CI) limits were estimated using univariate logistic regression in Stata version 15. Inter-observer agreement for radiographical features, including the *Brixia* score, were evaluated and expressed with the Kappa statistic. The agreement was classified as follows: perfect, Kappa = 0.81–1.0; substantial, Kappa = 0.61–0.80; moderate, Kappa = 0.41–0.60; fair, Kappa = 0.21–0.40; slight, Kappa = 0–0.21; no agreement, Kappa < 0.

The median number of days between the patient’s most severe X-ray and death was determined and interquartile ranges (IQR) were calculated.

### Ethical considerations

Ethical approval was obtained from the Biomedical Research Ethics Committee of the University of KwaZulu-Natal (BREC/00002590/2021) and site approval was obtained from the hospital. Informed consent was not required for this retrospective, descriptive study.

## Results

### Demographics

During the study period a total of 308 portable CXRs were performed on 197 inpatients with confirmed COVID-19 infection. Four patients were duplicates, and 18 patients under the age of 18 were excluded, resulting in a final study population of 175 patients for the analysis. Of the total number of CXRs performed, 111 X-rays were repeat X-rays, and despite not being included in the final study population, were evaluated for disease progression or regression. The mean age was 53.34 years, with an age range between 19 years and 98 years. The study population was subdivided into three age categories: 19–30 years; 31–60 years; and > 61 years. Twelve patients (6.9%) were in the 19–30 age group, with 106 patients (60.6%) in the 31–60 age group and 57 patients (32.6%) in the > 60 age group. Eighty-three patients (47.4%) were male, and 92 patients (52.6%) were female.

**FIGURE 2:**
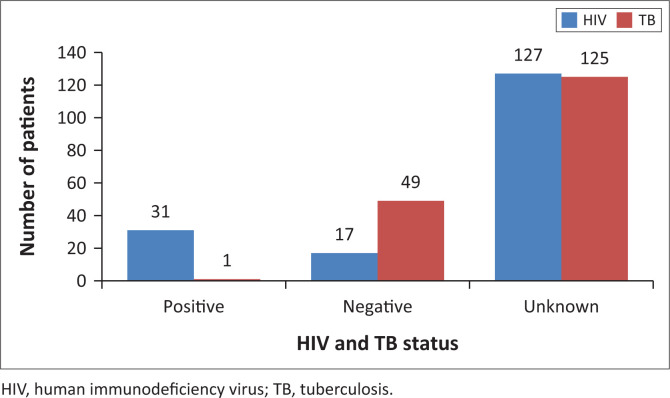
Graphical representation of patients with underlying human immunodeficiency virus and tuberculosis.

The HIV and TB statuses of the included participants is presented in Figure 2. According to the NHLS database, HIV infection was present in 17.70%, absent in 9.70% and unknown in 72.6%. TB infection was positive in 0.65%, negative in 28.00% and unknown in 71.40%.

### Radiological findings

Ground glass opacification (98.9%) was the most common CXR finding, followed by consolidation (86.3%), and pleural effusion (29.1%) (see Table 1). Involvement of bilateral lung fields (96.6%), no zonal predominance (61.7%) and 2–5 lobar involvement (99.4%) were the most frequent lung distribution patterns (see Table 1). Baseline CXRs were available in only 25 patients (14.3%) and 21 (84%) had a normal baseline X-ray. Multiple X-rays were performed on 97 patients (55.4%), and 56 of these (57.7%) demonstrated radiological disease progression. While 41 patients (42.3%) demonstrated disease regression.

**TABLE 1:** Summary of chest x-ray findings and patterns of disease.

Chest X-ray pattern	Present (*n* = 175)		Absent (*n* = 175)	
	*n*	%	*n*	%
Ground glass opacity	173	98.9	2	1.1
Consolidation	151	86.3	24	13.7
Bronchiectasis	7	4.0	168	96.0
Pleural effusion	51	29.1	124	70.9
Cavitation	4	2.3	171	97.7
Lymphadenopathy	1	0.6	174	99.4
Nodules/masses	0	0.0	175	100.0
Volume loss	15	8.6	160	91.4
Probable TB	7	4.0	168	96.0
Bilateral disease	169	96.6	6	3.4
Unilateral disease	2	1.1	173	98.9
Upper zone predominance	5	2.9	170	97.1
Lower zone predominance	61	34.9	114	65.1
No zonal predominance	108	61.7	67	38.3
1 lobe involved	1	0.6	174	99.4
2–5 lobes involved	174	99.4	1	0.6

TB, tuberculosis.

Quantification of the CXR findings using the *Brixia* score revealed moderate disease most frequently with a score between 13 and 16 (33.7%), followed by severe disease scoring between 16 and 18 (29.7%), less severe disease scoring between 7 and 12 (27.4%), and lastly mild disease scoring between 0 and 6 (9.1%). The Kappa statistic for inter-observer agreement was evaluated as substantial (Kappa = 0.64) for grading of CXR findings using the *Brixia* score.

### Disease outcomes

Of the final study population of 175, 118 patients (67.4%) were de-isolated into general wards or directly discharged from isolation, with the remaining 57 patients (32.6%) noted to have demised. Comparison of this outcomes data with the patient’s CXR *Brixia* score is summarised in Table 2.

**TABLE 2:** Summary of outcomes data and severity index using the *Brixia* scoring system.

*Brixia* score	Demised (*n* = 57)			De-isolated (*n* = 118)			Total (*n* = 175)		*p*	Odds ratio	95% CI
	*n*	% of respective *Brixia* group	% of total demised	*n*	% of respective *Brixia* group	% of total de- isolated	*n*	%			
0–6	1	6.3	1.8	15	93.7	12.7	16	9.2	-	-	
7–12	7	14.6	12.3	41	85.4	34.8	48	27.4	0.40	2.56	0.29–22.59
13–16	25	42.4	43.8	34	57.6	28.8	59	33.7	0.02	11.03	1.37–89.09
16–18	24	46.2	42.1	28	53.8	23.7	52	29.7	0.02	12.86	1.58–104.61

CI, confidence interval.

A pairwise comparison was performed when evaluating the *Brixia* score with outcome (Table 2). Using the lowest *Brixia* score 0–6 as a comparison reference, the percentage demised at 7–12 was an 8.3% increase. The OR was 2.56 indicating a relative increase in the odds of death, but this was not statistically significant (*p* = 0.40). Score group 13–16, and 16–18 demonstrated a statistically significant (*p* = 0.02 respectively) percentage increase (42.4% and 46.2% respectively) in the number of patients who demised.

**FIGURE 3:**
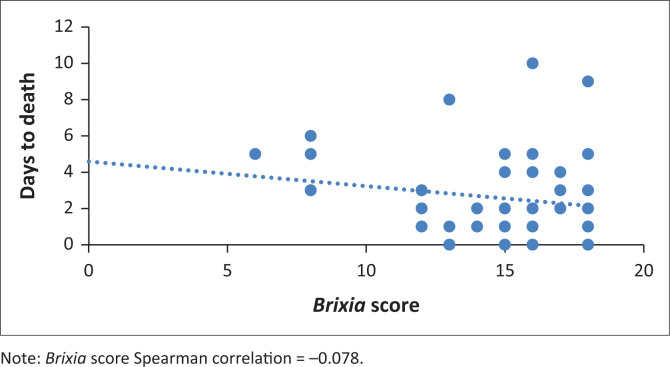
Graphical representation of days to death and Brixia score.

The number of days between death and the patient’s most severe CXR is outlined in Figure 3/Table 3. The median days between X-ray and death was lowest (1 day) in the *Brixia* score 13–16 group, followed by the 16–18 group (2 days), the 7–12 group (3 days), and the 0–6 group (5 days). However, these values were not deemed statistically significant with a *p*-value of 0.13.

**TABLE 3:** Number of days between X-ray and demise.

*Brixia* score	Total (*n* = 57)	Median	IQR	*p*
0–6	1	5	5–5	0.13
7–12	7	3	2–5	0.13
13–16	25	1	1–2	0.13
16–18	24	2	1–3	0.13

IQR, interquartile ranges.

## Discussion

The COVID-19 pandemic is an ongoing international public health emergency,^3^ with many countries now entrenched in the third and fourth waves of the disease. There are 3 004 203 confirmed cases, with 89 944 deaths recorded and 26 263 590 vaccine doses administered in South Africa as of 03 December 2021 (NICD). Chest imaging, particularly using computed tomography has proven beneficial in patients with early infection.^2^ Chest radiography has primarily been seen as a triage and adjuvant imaging tool.^12^

This study analysed the chest radiographic findings of confirmed COVID-19 patients admitted to a resource limited South African isolation hospital, where concomitant pulmonary manifestations of HIV and TB may pose an additional diagnostic dilemma. Unfortunately, the vast majority of our patient population had unknown laboratory confirmation of HIV and TB, which may be attributed to a number of factors, such as avoiding health facilities for voluntary counselling and testing (VCT) for HIV because of COVID-19, low confirmed TB as a result of initiation onto TB treatment on the basis of clinical TB symptoms and a suggestive CXR, and/or lastly confirmation of TB and HIV at other base hospitals.

**FIGURE 4:**
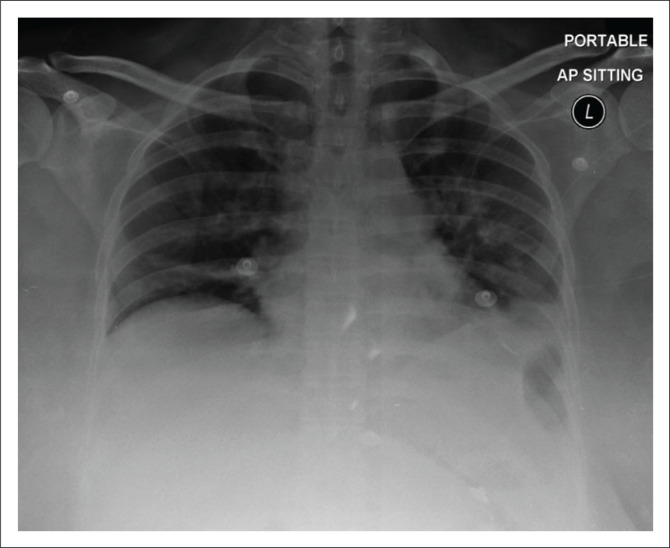
A 36-year-old female admitted to an isolation ward with COVID-19 pneumonia. Portable antero-posterior (AP) chest radiograph demonstrates blunting of the left costophrenic angle suggesting a pleural effusion as well as bilateral air space opacities. *Brixia* score: 13.

**FIGURE 5:**
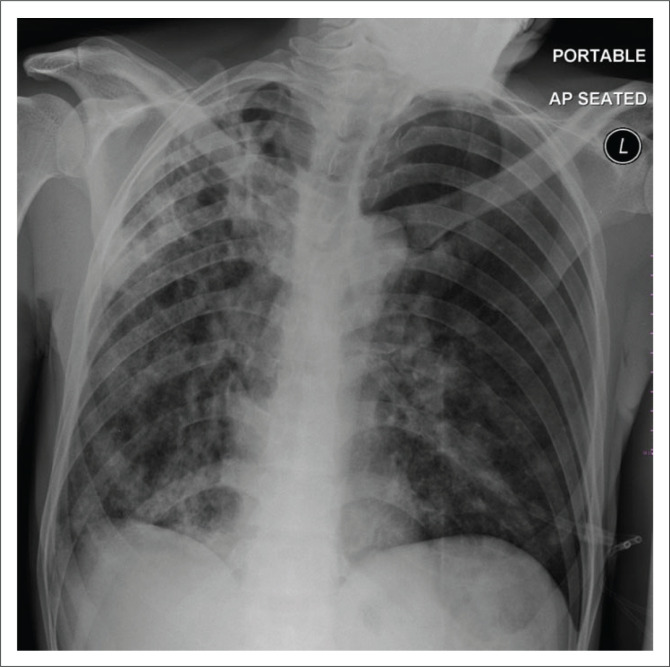
A 27-year-old male admitted to an isolation ward with COVID-19 pneumonia. Portable chest radiograph shows fibrosis and cavitation in the apical segment of the right upper lobe. Further bilateral right lung and left lingular air space opacities are noted. *Brixia* score: 15.

The most frequent CXR findings of COVID-19 pneumonia were ground glass opacification, followed by consolidation, and pleural effusion. Whilst the prevalence of ground glass opacification and consolidation are consistent with the literature, the frequency of pleural effusions (see Figure 4) is considered unusual; with Zhang et al. reporting pleural effusions mainly in cases of severe infection.^2,12^ The majority of the pleural effusions assessed were small in size, and were either considered reactive in aetiology or an indication of superadded infection. Cavitation (see Figure 5), lymphadenopathy, and bronchiectasis, which are findings that may be present in TB, were inconsistently reported in this study. This correlates to the low prevalence of confirmed TB as noted from the laboratory data in our study population and is thought to be a consequence of our relatively small sample size.

**FIGURE 6:**
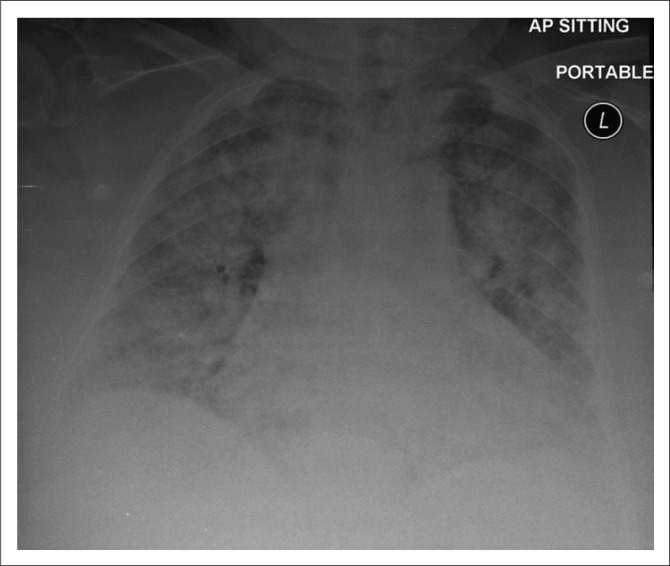
A 69-year-old female admitted to an isolation ward with COVID-19 pneumonia. Portable chest radiograph demonstrates air space opacification with no zonal predominance. *Brixia* score: 18.

**FIGURE 7:**
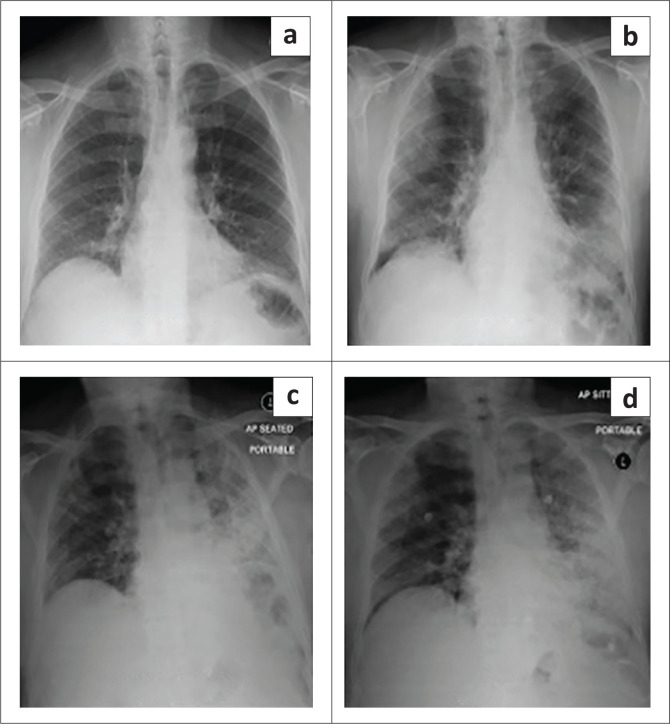
Chest radiographs from (a) 03 October 2013, (b) 27 June 2020, (c) 03 July 2020 and (d) 11 July 2020): 2013 normal baseline radiograph. Serial images demonstrate progressive unilateral left lower lobe and lingular air space opacification, consolidation and volume loss. Focal right upper lobe consolidation with ‘air bronchograms’.

In line with other available literature, the predominant disease distribution was a bilateral lung pattern with 2–5 lobes involved. Whilst other studies demonstrated lower zone predominance^12^; a diffuse disease process with no zonal predominance was most frequently seen (see Figure 6). A higher frequency of disease progression in the patients with multiple imaging (see Figure 7), was also noted in the studied sample. This may be attributed to the fact that the study population comprised exclusively admitted inpatients in which increased disease severity or rapid symptom progression was thought to necessitate admission.

Substantial inter-observer agreement was demonstrated when applying the *Brixia* CXR scoring system to the CXR findings. This is encouraging because of the large gap in experience levels between the observers and suggests that the *Brixia* score is an easily applicable quantification tool which may prove beneficial to clinicians without extensive radiological experience, especially where chest radiographs are more readily accessible than CT scans.

The study by Borghesi et al.^14^ examined the relationship between severity of lung disease, using the *Brixia* score and demographics like age and sex in a European population. Whilst limited literature is available in our unique South African setting, a study by du Bruyn et al.^16^ demonstrated that co-morbidities like TB co-exist with COVID-19 infection, and likely exacerbate each other. Whilst their study focused predominantly on clinical presentation and laboratory markers, the current study further examined the relationship between demographics, CXR findings with a severity scoring system, and outcome, in COVID-19 infection.

When comparing age and outcome, a borderline association was evident, with younger patients (between 19 and 30 years) more likely to recover, and older patients (> 60 years) at greater risk of death in comparison to their respective counterparts. A relatively even distribution of outcomes was noted between males and females and there was no significant association between sex and outcome.

In the 16–18 *Brixia* score range, the percentage of patients who demised as a subgroup of this group was 46.2%, more than seven times the percentage of the 0–6 group and was found to be statistically significant at *p* = 0.02. This implies that the risk of death from COVID-19 infection is more than twelve times greater if you have a severity score (*Brixia* score) of 16–18, compared to those with a milder imaging severity (score group of 0–6). This relationship between lung severity scoring and outcome is validated by clinical outcomes, as patients with low *Brixia* scores are likely expected to recover, whilst those with high severity indices are at higher risk for mortality.

The highest frequency of overall patients that demised had *Brixia* scores between 13 and 16, followed by the 16–18 *Brixia* range. A possible explanation for this is that the 13–16 group had the highest frequency of patients overall, despite their worst CXR (and thus highest *Brixia* severity score) being used for analysis.

## Study limitations

The limitations of this study include its retrospective study design and relatively small sample size. This was also a single centre study; therefore, patients admitted to other facilities were not included. Furthermore, only inpatients with confirmed COVID-19 disease were included.

A lack of analysis and comparison between co-morbidities like HIV and TB and CXR findings was also a limiting factor, which is attributed to relevant laboratory data being available for only a small percentage of patients.

Lack of knowledge of symptom onset was another limiting factor. The X-rays evaluated were taken at an unknown time during the patients COVID-19 disease timeline, and if the X-ray was performed early in disease, and no later X-ray was performed, the *Brixia* score and consequently the correlation between the *Brixia* score and outcome may have been underestimated.

Finally, only a portable X-ray unit was used to obtain CXRs, as all patients with COVID-19 were admitted to isolation wards. Elements of poor inspiration, rotation, and positioning (supine versus seated) likely limit the diagnostic accuracy of CXR interpretation.

## Conclusion

When evaluating chest radiography in patients with COVID-19 infection, features of ground glass opacification, consolidation and pleural effusions, with bilateral lung involvement and no zonal predominance were most frequently found. Thus, the radiological presence of these features must raise awareness of suspected COVID-19 infection. Whilst only a small percentage of patients in our study had laboratory confirmed or radiological evidence of co-morbid conditions like HIV and TB, further studies with larger sample sizes are required to assess the impact that the pulmonary manifestations of these conditions have on the CXR findings of COVID-19 pneumonia in the South African setting.

Of note, the application of a severity index on an easily accessible imaging modality such as a CXR, the *Brixia* scoring system, can be used to prognosticate COVID-19 disease severity. It seemingly requires minimal clinician training, and may assist with timeous assessment of the disease severity in COVID-19 positive patients, as an overall indicator of clinical outcome, suggesting the use of early intervention strategies to improve overall patient prognosis.
